# Genome-Wide Analysis Identifies *IL-18* and *FUCA2* as Novel Genes Associated with Diastolic Function in African Americans with Sickle Cell Disease

**DOI:** 10.1371/journal.pone.0163013

**Published:** 2016-09-16

**Authors:** Julio D. Duarte, Ankit A. Desai, Justin R. Sysol, Taimur Abbasi, Amit R. Patel, Roberto M. Lang, Akash Gupta, Joe G. N. Garcia, Victor R. Gordeuk, Roberto F. Machado

**Affiliations:** 1 Department of Pharmacy Practice, University of Illinois at Chicago, Chicago, IL, United States of America; 2 Division of Cardiology, Sarver Heart Center, University of Arizona, Tucson, AZ, United States of America; 3 Department of Medicine, University of Illinois at Chicago, Chicago, IL, United States of America; 4 Department of Medicine, Mercy Hospital and Medical Center, Chicago, IL, United States of America; 5 Department of Medicine, University of Chicago, Chicago, IL United States of America; 6 Department of Medicine, University of Arizona, Tucson, AZ, United States of America; Universidad de Granada, SPAIN

## Abstract

**Background:**

Diastolic dysfunction is common in sickle cell disease (SCD), and is associated with an increased risk of mortality. However, the molecular pathogenesis underlying this development is poorly understood. The aim of this study was to identify a gene expression profile that is associated with diastolic function in SCD, potentially elucidating molecular mechanisms behind diastolic dysfunction development.

**Methods:**

Diastolic function was measured via echocardiography in 65 patients with SCD from two independent study populations. Gene expression microarray data was compared with diastolic function in both study cohorts. Candidate genes that associated in both analyses were tested for validation in a murine SCD model. Lastly, genotyping array data from the replication cohort was used to derive cis-expression quantitative trait loci (cis-eQTLs) and genetic associations within the candidate gene regions.

**Results:**

Transcriptome data from both patient cohorts implicated 7 genes associated with diastolic function, and mouse SCD myocardial expression validated 3 of these genes. Genetic associations and eQTLs were detected in 2 of the 3 genes, *FUCA2* and *IL18*.

**Conclusions:**

*FUCA2* and *IL18* are associated with diastolic function in SCD patients, and may be involved in the pathogenesis of the disease. Genetic polymorphisms within the *FUCA2* and *IL18* gene regions are also associated with diastolic function in SCD, likely by affecting expression levels of the genes.

## Introduction

Sickle cell disease (SCD) is a heritable hemolytic anemia caused by a mutation of β-globin chain of hemoglobin. This mutation results in sickle hemoglobin (HbS), which leads to hemolysis, increased blood viscosity, abnormal interactions with the vascular environment, and chronic damage to multiple organ systems including the heart.[1] While gross systolic or contractile dysfunction is rare (such as reduced left ventricular ejection fraction, diastolic dysfunction, or abnormal relaxation of the myocardium), diastolic dysfunction is a common cardiac complication in patients with SCD and is thought to stem from multiple causes, including systemic vasculopathy (which affects afterload), myocardial fibrosis, and a compensatory hyperdynamic state due to anemia.[2] Diastolic dysfunction in this population is also associated with aortic stiffness, mildly elevated systolic blood pressure, and decreased oxygen saturation.[3–5]

Defining diastolic dysfunction via non-invasive transthoracic echocardiographic (TTE) or invasive hemodynamics (right heart catheterization or RHC) is challenging in patients with sickle cell disease, as their high output state can impact myocardial function and responses. Therefore, the American Society of Echocardiography guidelines for defining diastolic dysfunction or “normal” ranges for wedge pressures may not necessarily be exactly applicable in patients with sickle cell disease. Evidence of this observation was apparent in a reported sickle cell registry.[2] In this analysis, echocardiographic-based values for ratios of mitral valve inflow (E) velocities over tissue Doppler mitral valve velocities (e′) were correlated with invasive measurements of left ventricular filling pressures. However, the E/e′ ranges that defined elevated filling pressures were significantly different from what has been published for the general (or non-sickle) population. Nonetheless, increased E/e′ has been associated with decreased functional capacity and mortality and, in SCD patients, E/e′ > 8.2 is considered a reasonable surrogate for diastolic dysfunction.[2, 6, 7]

Defining the molecular pathogenesis of this manifestation is therefore important given a 3.5-fold increased risk of mortality in patients with an elevated E/e’ in SCD.[8] Thus, despite potential differences in ranges, echocardiographic studies indicate that in SCD, the E/e′ ratio can help define diastolic dysfunction.[2, 6] Improved mechanistic understanding of diastolic dysfunction could lead to better diagnostic markers of SCD-associated diastolic dysfunction and the ability to potentially predict its development before it progresses.

The aim of this study was to identify candidate genes that are associated with diastolic function in SCD, potentially helping to elucidate the molecular mechanisms behind its development as well as informing prediction of diastolic dysfunction in patients with SCD. Herein, we report the discovery of novel candidate genes associated with diastolic function in a SCD patient population, which were derived from two independent SCD patient populations, and experimentally validated in a mouse SCD model. Further, we identify genetic polymorphisms within these genes that are associated with diastolic function and serve as expression quantitative trait loci (eQTLs).

## Methods

### Patient Population

Adult SCD patients were recruited from outpatient clinics at the University of Chicago (discovery cohort) and the University of Illinois at Chicago (replication cohort). All subjects provided informed written consent to participate in this study with the approval by the respective institutional review boards (University of Illinois at Chicago IRB and University of Chicago IRB).

The discovery cohort consisted of 29 clinically stable African American subjects with homozygous SCD (HbSS demonstrated by high-performance liquid chromatographic separation or gel electrophoresis) recruited from the University of Chicago. In this cohort, subjects underwent prospective transthoracic echocardiogram (TTE) as part of a research protocol regardless of clinical symptoms on the same day as phlebotomy for gene expression profiling. The replication cohort consisted of 32 African American subjects with SCD (28 with HbSS, 3 with HbSC, and 1 with HbSβ-thalassemia genotype, demonstrated by high-performance liquid chromatographic separation or gel electrophoresis) from the University of Illinois at Chicago. TTE studies completed as part of clinical care were read retrospectively by a board-certified cardiologist following American Society of Echocardiography guidelines. Subjects from all centers were excluded if they were clinically unstable, defined by having vaso-occlusive crisis, acute chest syndrome or unscheduled blood transfusions within 3 weeks of the study. Details of subject recruitment, and TTE measurements for both of these cohorts have been described previously.[5, 9, 10]

### Microarray Analysis

RNA was isolated from peripheral blood mononuclear cells using the RNeasy Plus Kit (Qiagen, Valencia, CA, USA) in the discovery cohort and Trizol (Invitrogen, Carlsbad, CA) in the replication cohort according to the manufacturer’s protocol. The discovery cohort underwent microarray gene expression analysis using the Human Exon 1.0 ST Array (Affymetrix, Santa Clara, CA, USA), while the replication cohort used the newer Human Gene 2.0 ST Array (Affymetrix). Details of RNA isolation, target labeling, hybridization, and scanning of arrays in both the discovery [9] and validation [10] cohorts have been previously outlined. Expression data have been submitted to the Gene Expression Omnibus (accession #: GSE38528). Quality control and RMA normalization of raw data files were completed using the “oligo” package for R version 3.1.1.[11] Only probeset IDs with transcript annotation information from Affymetrix were included in the gene expression analyses.

Previous data indicate that an E/e′ value of greater than 8.2 has adequate positive predictive value for detecting right heart catheterization-derived elevated pulmonary capillary wedge pressure (PCWP) in SCD patients—a marker for diastolic dysfunction in SCD.[2, 6] Associations between normalized gene expression values and lateral and septal E/e′ was performed using both Pearson’s correlation test and, to confirm that potential confounders did not affect associations, linear regression, adjusting for age, sex, and hydroxyurea use. In the discovery cohort, genes with expression values that associated with E/e′ exhibiting a *P*-value ≤ 0.05 were similarly tested in the replication cohort. In order to be considered a true replication, correlations were required to associate in the replication cohort with the same direction of effect and *P* ≤ 0.05. To further validate diastolic function associations, we performed association analyses of all filtered candidate genes with PCWP in a subset of the replication cohort that underwent right heart catheterization (RHC, N = 23) using Pearson’s correlation test (*P* ≤ 0.05 was considered significant). Candidate gene were chosen for further study if they met at least one of the following two criteria: (1) replication of E/e′ associations from the discovery cohort in the replication cohorts with a combined *P* ≤ 0.0001 (using Fisher's combined probability test); (2) similar correlation (*P* ≤ 0.05) with E/e′ in both cohorts and similar association with PCWP (*P* ≤ 0.05) in the replication cohort. All statistical analyses were completed using R version 3.1.2.[12]

### Gene Expression Analysis in Murine Myocardial Tissue

Sickle cell “Townes” strain mice (Jackson Laboratory, Stock No: 013071) were grown for at least seven months. Left ventricular myocardial tissue was isolated from both control (βA/βA) and sickle cell (βS/βS) mice (N = 5–7 per group). Total RNA from the snap-frozen myocardial tissue was isolated using the RNeasy Plus Mini Kit (Qiagen). Two micrograms of purified RNA was reverse transcribed to cDNA using the High Capacity cDNA Reverse Transcription kit (Applied Biosystems). Real-time polymerase chain reaction (RT-PCR) was performed using Taqman quantitative RT-PCR assays on a CFX384 Real-Time PCR Detection System (Bio-Rad). Relative mRNA expression levels of *Adam9*, *Fuca2*, *Il18*, *Olfr658* (closest murine equivalent to *OR52N4*), *Prosc*, *Slc16a2*, and *Sult2b1* were normalized to the expression of glyceraldehyde 3-phosphate dehydrogenase (*Gapdh*) and determined by the ΔΔCt method, according to manufacturer’s guidelines. Differences in gene expression between sickle cell and control tissue were calculated using Students t-test, with a *P*-value ≤ 0.05 considered significant. Significant differences in gene expression with similar directional effect as observed in the clinical SCD cohorts were considered validations.

### Transthoracic Echocardiography (TTE)

TTE was performed in both control (βA/βA, N = 4) and sickle cell (βS/βS, N = 10) mice using a Vevo 2100 High Resolution Imaging System (Visual-Sonics, Toronto, Canada) with an MS550D scan head designed for murine cardiac imaging. Following anesthetic induction in 3% isoflurane, mice were placed in a supine position on a heated platform for echocardiography. Body temperature was maintained at 37°C and anesthesia was maintained with 1.5% isoflurane (USP, Phoenix, AZ) in 100% oxygen. Imaging was performed at a depth setting of 1 cm. Images were collected and stored as digital cine loops for off-line calculations. Standard imaging planes, M-mode, Doppler, and functional calculations were obtained according to American Society of Echocardiography guidelines [13, 14]. Doppler imaging was obtained from an apical 4-chamber view to assess LV filling and tissue velocity of the septal mitral valve annulus.

### Genetic Association and eQTL Analysis

DNA was isolated from patient blood samples in the replication cohort and genotyped using the Axiom Pan-African Genotyping Array (Affymetrix) as described previously.[10] Single nucleotide polymorphisms (SNPs) with a minor allele frequency ≥ 0.05 located within 10 kb of the candidate genes previously validated in murine myocardial tissue were selected for eQTL analysis. Selected SNPs were compared to expression of *FUCA2*, *IL18*, and *SLC16A2* using linear regression, adjusting for age, sex, and hydroxyurea use. Similarly, these same SNPs were directly tested for association with septal and lateral E/e′. All analyses were completed in PLINK 1.9,[15] and a *P*-value ≤ 0.05 was considered significant.

## Results

Overall, demographics and clinical characteristics of the discovery and replication cohorts were similar in both groups of patients, those with elevated E/e’ and those with normal filling pressures (Table 1). In the replication cohort, patients with elevated E/e’ tended to be older and have a higher systolic blood pressure, while these characteristics were not significantly different in the discovery population (Table 1).

**Table 1:** Patient characteristics within SCD discovery and replication cohorts.

Characteristic	Discovery			Replication		
	Elevated E/e’ (*N* = 18)	Normal E/e’ (*N* = 11)	*P*	Elevated E/e’ (*N* = 17)	Normal E/e’ (*N* = 14)	*P*
Age (years ± SD)	32.1 ± 8.5	31.5 ± 7.3	0.85	41.4 ± 11.5	27.5 ± 7.1	0.002
Male (%)	33	50	0.41	50	57	0.68
Hgb SS genotype (%)	94	90	0.67	87.5	90	0.64
BSA (m^2^)	1.75 ± 0.17	1.77 ± 0.19	0.86	1.85 ± 0.2	1.76 ± 0.1	0.72
SBP (mmHg)	118 ± 23.5	116 ± 6.2	0.80	135 ± 11.8	110 ± 10.9	0.006
DBP (mmHg)	65 ± 19.6	64 ±6.2	0.95	71.5 ± 13.1	62 ± 7.1	0.33
LA Volume/BSA	67.3 ± 16.9	67.4 ± 21.8	1	43.0 ± 12.7	51.3 ± 16.1	0.17
LVEF (%)	58.6 ± 6.1	60.4 ± 7.3	0.53	54.9 ± 7.7	58.6 ± 8.8	0.26
TRV (m/s)	2.41 ± 0.46	2.23 ± 0.35	0.35	2.24 ± 0.69	2.02 ± 0.38	0.88
Hydroxyurea Therapy (%)	33	50	0.73	36	40	0.76
Hgb (g/dL)	7.71 ± 1.20	8.78 ± 1.5	0.06	8.11 ± 1.7	8.65 ± 1.3	0.35
Serum Creatinine (mg/dL)	0.94 ± 0.61	0.64 ± 0.15	0.17	1.58 ± 1.9	0.81 ± 0.6	0.16
>2 lifetime blood transfusions (%)	72	70	0.91	86	93	0.55

SD: standard deviation; Hgb: hemoglobin; BSA: body surface area; SBP: systolic blood pressure; DBP; diastolic blood pressure; LA: left atrial; LVEF: left ventricular ejection fraction; TRV: tricuspid regurgitant velocity.

### Differential Gene Expression Associated with Diastolic Function

In the discovery cohort, 12,549 transcripts were obtained. In the replication cohort, 29,605 annotated transcripts were available given the different platform used. Twenty-eight transcripts that were correlated with E/e′ in the discovery cohort were also associated in the replication cohort with a similar direction of effect (S1 File). Of those, four genes (*FUCA2*, *ADAM9*, *IL18*, and *PROSC*) associated with E/e′ in both cohorts with a combined *P*-value ≤ 0.0001, and three genes (*OR52N4*, *SULT2B1*, and *SLC16A2*) associated with E/e′ in both cohorts as well as PCWP in the replication cohort (Table 2). Adjusting for covariates using linear regression yielded similar associations for *FUCA2* (combined *P* = 1.7 x 10^−6^), *ADAM9* (combined *P* = 2.8 x 10^−6^), *IL18* (combined *P* = 5.0 x 10^−5^), *PROSC* (combined *P* = 6.1 x 10^−5^), *OR52N4* (combined *P* = 0.0023), *SULT2B1* (combined *P* = 0.0004), and *SLC16A2* (combined *P* = 0.0021). These seven genes that met the selection criteria were designated for further validation as candidates associated with diastolic function.

**Table 2:** Candidate genes associated with diastolic function in SCD discovery and replication cohorts.

Gene	Gene Name	Associated Phenotype	Correlation coefficient		Combined *P*	PCWP (replication cohort)	
			Discovery	Validation		Correlation coefficient	*P*
*FUCA2*	Fucosidase, Alpha-L- 2, Plasma	Lateral E/e′	0.451	0.631	2.3 x 10^−6^	-0.299	0.271
*ADAM9*	ADAM Metallopeptidase Domain 9	Lateral E/e′	0.442	0.490	9.5 x 10^−5^	-0.153	0.484
*IL18*	Interleukin 18	Lateral E/e′	0.447	0.467	0.0001	0.190	0.385
*PROSC*	Proline Synthetase Co-Transcribed Homolog	Lateral E/e′	0.454	0.457	0.0001	-0.214	0.326
*OR52N4*	Olfactory Receptor, Family 52, Subfamily N, Member 4	Septal E/e′	-0.377	-0.433	0.0007	-0.434	0.038
*SULT2B1*	Sulfotransferase Family, Cytosolic, 2B, Member 1	Septal E/e′	0.418	0.385	0.0009	0.403	0.050
*SLC16A2*	Solute Carrier Family 16, Member 2 (Thyroid Hormone Transporter)	Septal E/e′	0.384	0.378	0.0016	-0.472	0.023

### Experimental Validation of Candidate Genes in a Pre-Clinical SCD Mouse Model

**Fig 1:**
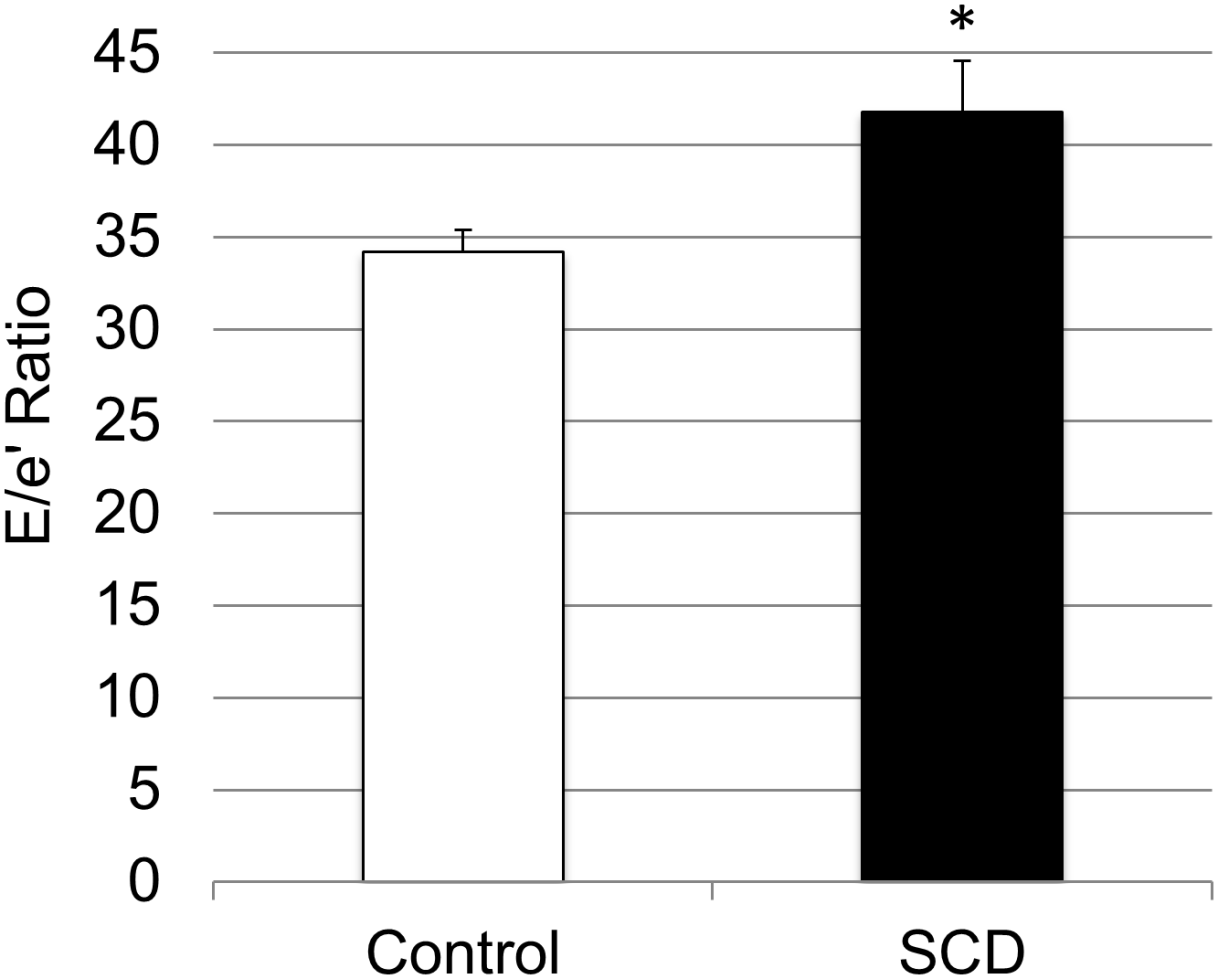
Comparison of E/e′ ratio between mice with and without sickle cell disease. Error bars denote standard error of the mean. * ≤ 0.05.

**Fig 2:**
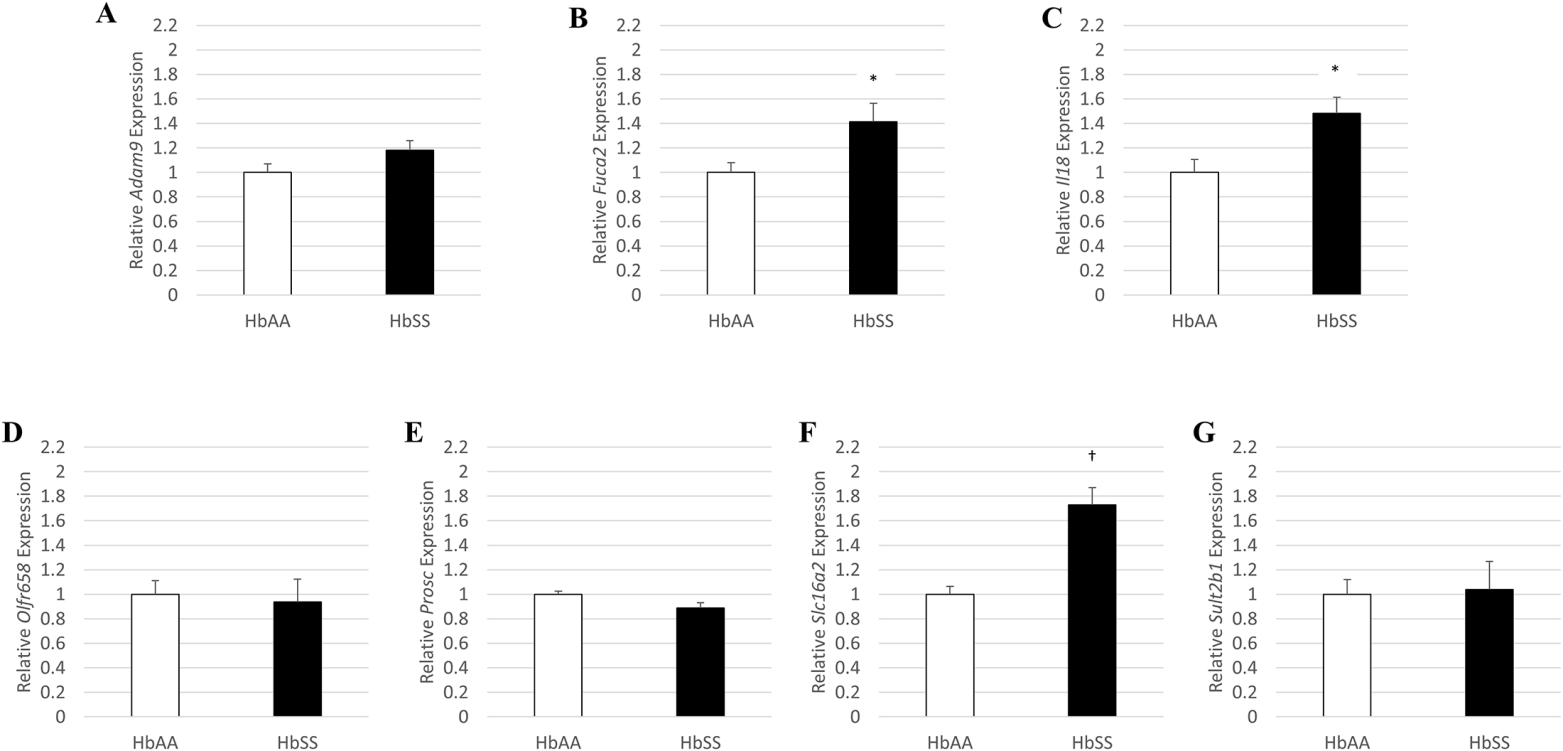
Comparison of candidate gene expression levels in myocardial tissue of mice with and without sickle cell disease. A: *Adam9*, B: *Fuca2*, C: *Il18*, D: *Olfr658*, E: *Prosc*, F: *Slc16a2*, G: *Sult2b1*. Error bars denote standard error of the mean. * ≤ 0.05; † ≤ 0.001.

It has been previously established that mice with SCD develop diastolic dysfunction by two months of age.[16] We confirmed that SCD mice have significantly higher E/e′ ratios than their control counterparts (Fig 1, *P* = 0.024). We then compared expression levels of the seven candidate genes between left ventricular myocardial tissue of mice with SCD and without SCD. Compared to control mice, SCD mice had significantly higher myocardial expression of *Fuca2* (*P* = 0.024), *Il18* (*P* = 0.025), and *Slc16a2* (*P* = 0.0004; Fig 2). These expression levels were directionally consistent with findings within the clinical discovery and replication cohorts, and thus were selected for further evaluation for eQTL analysis. Expression levels of *Adam9*, *Olfr658*, *Prosc*, and *Sult2b1* were not significantly different between SCD and control mice (Fig 2).

### Genetic Association and eQTLs within Candidate Gene Regions

In order to begin exploring the potential mechanism by which expression of *FUCA2*, *IL18*, and *SLC16A2* are altered in diastolic dysfunction, cis-eQTL testing was completed. From the 40 total SNPs identified within the remaining three candidate gene regions (no SNPs were available in the *SLC16A2* gene region), five SNPs in *FUCA2* and *IL18* were associated with expression of their respective genes (Table 3). To confirm their effect on diastolic function in SCD, these five SNPs were also tested for association with E/e′, and rs11214107 (in *IL18*; *P* = 0.030) and rs9496646 (in *FUCA2*; *P* = 0.003) were also significantly associated with E/e′ with a consistent direction of effect as that observed in the eQTL analysis (Table 3). To test whether the eQTL associations we observed were consistent in human myocardial tissue, we also analyzed the SNP-gene pairs using GTEx [17] left ventricular tissue data. Only rs8161 in *FUCA2* replicated as an eQTL (*P* = 0.015) in the GTEx analysis (Table 3).

**Table 3:** Cis-eQTL and genetic associations with E/e′ within the *IL18* and *FUCA2* gene regions.

SNP	Gene	SCD eQTL		Lateral E/e′		GTEx eQTL	
		β	*P*	β	*P*	β	*P*
rs11214107	*IL18*	0.368	0.045	4.343	0.030	0.10	0.43
rs9496646	*FUCA2*	0.301	0.001	2.626	0.003	-0.12	0.015
rs8161	*FUCA2*	-0.240	0.007	-1.589	0.076	-0.12	0.015
rs13197626	*FUCA2*	-0.289	0.008	-0.6028	0.58	0.096	0.051
rs6915645	*FUCA2*	0.276	0.036	0.4356	0.7432	-0.050	0.33

## Discussion

Although the increased mortality risk associated with diastolic dysfunction in SCD patients was reported nearly a decade ago,[8] the mechanistic underpinnings behind its development remain poorly understood. From humans and pre-clinical animal models, the role of fibrotic deposition in the myocardium (with its eventual impact on cardiac function) and the development of diastolic dysfunction have been previously observed.[2] Herein, we report that three genes, interleukin-18 (*IL18*), α-L-fucosidase A2 (*FUCA2*), and thyroid hormone transporter (*SLC16A2*), appear to be upregulated in SCD-associated diastolic dysfunction. We initially observed association between these genes and E/e′ (an established indicator for diastolic dysfunction) in a clinical SCD population, which we then replicated in an independent SCD population. We subsequently found that these genes were also upregulated in left ventricular tissue of mice with SCD. Moreover, SNPs within *IL18* and *FUCA2* gene regions were both associated (*SLC16A2* was not able to be tested) with diastolic dysfunction as well as expression of their respective gene. To our knowledge, this is the first report of these genes being associated with myocardial diastolic function in SCD.

Interleukin 18 (IL-18) is a well-characterized pro-inflammatory and fibrotic cytokine in the IL-1 family. Increased circulating levels of IL-18 are associated with development of heart failure and cardiac hypertrophy, as well as increased mortality in HF patients.[18, 19] In addition, IL-18 is associated with decreased left ventricular contractility and myocardial relaxation in both mice and humans, possibly via p38 MAPK.[20, 21] Most recently, inhibition of IL-18 has been reported to inhibit the development of diastolic heart failure in a mouse model.[22] The fact that IL-18, a cytokine with established pathophysiological relevance to diastolic dysfunction, was one of the three genes that were validated reinforces the potential biologic relevance of our candidate gene list in SCD.

Encoded by the highly homologous *FUCA1* and *FUCA2*, α-L-fucosidases catalyze the removal of terminal l-fucose residues linked via α-1,2, α-1,3, α-1,4 or α-1,6 to the reducing end of *N*-acetyl glucosamine of oligosaccharide chains. This enzyme is found both in the lysosome as well as on the outer plasma membrane.[23] In fact, fucoglycoconjugates are involved in a variety of physiological processes, such as immune response, signal transduction, early embryogenesis and development, apoptosis, adhesion of pathogens, and extravasation of leukocytes. Altered expression of fucosylated glycans have also been observed in several pathological processes, including atherosclerosis, sperm maturation in mammals, and cancer. In addition, increased α-l-fucosidase activity in serum is a marker of the development of colorectal and hepatocellular carcinomas.[24] Loss-of-function mutations in *FUCA1* have been associated with fucosidosis, a potentially fatal disease leading to a lethal accumulation of fucosylated glycosphingolipids,[25] but it is not clear if loss of *FUCA2* function yields a similar phenotype. Given its pleiotropic role, we speculate that the association of increased *FUCA2* expression associated with diastolic dysfunction may represent a novel potential role in the heart.

Thyroid hormone can modulate many aspects of the cardiovascular system, including cardiac contractility, heart rate, blood pressure, and systemic vascular resistance.[26] While hyperthyroidism is associated with increased cardiac heart rates and hypertrophy,[27, 28] hypothyroidism is associated with heart failure with slowing of the isovolumic relaxation phase of diastolic function and often accompanied by a rise in diastolic blood pressure with a sodium sensitive form of hypertension.[26] *SLC16A2* encodes the high-affinity thyroid hormone transporter MCT8, which is expressed in multiple organs, including the heart and is responsible for transporting thyroid hormone into the cell. A missense mutation in human MCT8 has been reported, leading to consistently elevated T3 levels and partially recapitulates a hyperthyroidism phenotype including persistent tachycardia, which reduces to a normal heart rate with treatment.[29] We can then speculate that over-expression of MCT8, present in myocardium of SCD mice, may modify opposing effects with a phenotype similar to hypothyroidism, with further exacerbation of diastolic blood pressures and function in the myocardium as is evident in a subset of patients with SCD.

Further confirming our gene expression data, SNP associations were also observed within the candidate gene regions of *IL18* and *FUCA2*. Because no SNPs were available to within the *SLC16A2* gene region, we were unable to test cis-eQTLs and genetic associations within this gene. Our genetic association data suggest that the variability in *IL18* and *FUCA2* expression may be the result of cis-eQTLs. Notably, we observed that rs8161 is an eQTL in both PBMCs (from our data) and in LV tissue (GTEx data), as well as nominally associated with lateral E/e′. However, *in silico* review of all discovered eQTLs did not reveal obvious mechanisms by which they would exert control on gene expression. These SNPs could either be in an unknown regulator region (such as a repressor or enhancer) or perhaps be in linkage disequilibrium with another functional SNP. Additional genotyping in these gene regions coupled with functional studies are needed to elucidate the precise role of variation in these gene region in development of SCD-associated diastolic dysfunction.

Our study has several limitations. First, estimation of diastolic dysfunction using non-invasive methods such as E/e′ is not ideal. However, clinical data utilizing E/e′ have still shown significant correlations with mortality in sickle cell disease,[8] indicating the measure possesses reasonable robustness.

Second, the *P*-value thresholds used in our screening for candidate genes associated with diastolic function were liberal. Because two independent research populations were available, we were able to take advantage of a replication strategy (planned *a priori*) rather than relying on a single, stringently-adjusted *P*-value to filter out false positives. Using such a *P*-value threshold would almost certainly increase the risk of discarding true associations with weaker *P*-values due to a difficult to define phenotype and a relatively small sample size. On the other hand, the low-stringency thresholds used in our screening of our discovery and replication cohorts created a considerable risk of false positives being incorporated to the candidate gene list. Thus, we decided to further validate our clinical findings within mouse myocardial tissue. This analysis provided the additional benefit of correlating clinically-observed gene expression in PBMCs with myocardial expression to assure that these gene expression changes were consistent in the LV. In fact, the probability of a gene associating in all of the expression analyses, with the same direction of effect, is actually smaller (3.1 x 10^−6^) than the probability threshold from a Bonferroni adjustment (4.0 x 10^−6^) in our patient analysis (S1 file).

Third, because we report disease-gene associations, we cannot infer causality with any of the reported genes, making their role in disease development hypothetical. However, *FUCA2*, *IL18*, and *SLC16A2* are unlikely to simply be genomic markers for worsening SCD because biomarkers (such as hemoglobin, hydroxyurea use, and number of transfusions) indicate that SCD severity was not different between those with and without diastolic dysfunction (E/e′ > 8.2) in both cohorts (Table 1). Additional functional studies are needed to confirm that *FUCA2*, *IL18*, and *SLC16A2* are involved in development of diastolic dysfunction in SCD, rather than simply markers of deteriorating diastolic function.

In conclusion, we have discovered consistent associations between diastolic function and *FUCA2*, *IL18*, and *SLC16A2*, which we confirmed were also differentially expressed in the myocardium of SCD mice. Further, we found that SNPs within the gene regions of *FUCA2* and *IL18* also associate with both diastolic function and expression levels of their respective genes. While these data make a compelling case that these genes are potentially involved in the pathogenesis of diastolic dysfunction in SCD, the manner in which they are involved still remains to be determined.

## Supporting Information

S1 File E/e′ and PCWP associations and probability of finding associations by chance through replication. Transcripts in italics met the criteria for validation in a murine SCD model.
(DOCX)

S2 File Clinical genotype and murine cardiovascular data. SCD patient genotypes; murine E/e′ measurements; murine candidate myocardial gene expression values.
(XLSX)
