# Supplementary material for: Genome-Wide Analysis Identifies IL-18 and FUCA2 as Novel Genes Associated with Diastolic Function in African Americans with Sickle Cell Disease
Source: PLoS One. 2016 Sep 16;11(9):e0163013. doi: 10.1371/journal.pone.0163013 (PMC5026353; doi:10.1371/journal.pone.0163013)
Supplement: S1 File — Transcripts in italics met the criteria for validation in a murine SCD model. (DOCX) [file pone.0163013.s001.docx]

**Genome-wide analysis identifies *IL-18* and *FUCA2* as novel genes associated with diastolic function in sickle cell disease**

Julio D. Duarte, Ankit A. Desai, Justin R. Sysol, Taimur Abbasi, Amit R. Patel, Roberto M. Lang, Akash Gupta, Joe G. N. Garcia, Victor R. Gordeuk, and Roberto F. Machado

**Supplementary Material**

| **Supplementary Table 1: E/eʹ and PCWP associations in discovery and validation cohorts** | | | | | | | | |
| --- | --- | --- | --- | --- | --- | --- | --- | --- |
| **Transcript** | **Phenotype** | **Correlation Coefficient (Discovery)** | ***P* (Discovery)** | **Correlation Coefficient (Validation)** | ***P* (Validation)** | **Combined *P*** | **PCWP Correlation Coefficient (Validation)** | **PCWP *P* (Validation)** |
| *ADAM9* | *Lateral E/eʹ* | *0.442* | *0.018* | *0.490* | *0.005* | *8.84E-06* | -0.154 | 0.484 |
| AIMP2 | Lateral E/eʹ | 0.482 | 0.009 | 0.372 | 0.039 | 3.73E-05 | -0.220 | 0.312 |
| ALDH2 | Lateral E/eʹ | 0.393 | 0.039 | 0.422 | 0.018 | 7.26E-05 | -0.251 | 0.248 |
| ARRDC4 | Lateral E/eʹ | 0.383 | 0.044 | 0.378 | 0.036 | 1.77E-04 | -0.180 | 0.411 |
| CHMP2A | Lateral E/eʹ | 0.426 | 0.024 | 0.423 | 0.018 | 4.32E-05 | 0.105 | 0.532 |
| COPS6 | Lateral E/eʹ | 0.396 | 0.037 | 0.371 | 0.040 | 1.64E-04 | -0.309 | 0.151 |
| CREBL2 | Lateral E/eʹ | 0.477 | 0.010 | 0.405 | 0.024 | 2.40E-05 | -0.097 | 0.661 |
| EIF3K | Lateral E/eʹ | 0.415 | 0.028 | 0.420 | 0.019 | 5.40E-05 | -0.143 | 0.515 |
| EIF3L | Lateral E/eʹ | 0.410 | 0.030 | 0.389 | 0.031 | 9.86E-05 | -0.431 | 0.040 |
| FTSJ2 | Lateral E/eʹ | 0.395 | 0.038 | 0.394 | 0.028 | 1.15E-04 | -0.075 | 0.735 |
| *FUCA2* | *Lateral E/eʹ* | *0.451* | *0.016* | *0.631* | *0.0001* | *1.76E-07* | -0.239 | 0.271 |
| GLYR1 | Lateral E/eʹ | 0.385 | 0.043 | 0.364 | 0.044 | 2.12E-04 | -0.108 | 0.624 |
| GNA12 | Septal E/eʹ | 0.416 | 0.028 | 0.408 | 0.023 | 6.48E-05 | -0.233 | 0.284 |
| *IL18* | *Lateral E/eʹ* | *0.447* | *0.017* | *0.467* | *0.008* | *1.29E-05* | -0.253 | 0.243 |
| METTL9 | Lateral E/eʹ | 0.443 | 0.018 | 0.432 | 0.015 | 2.78E-05 | -0.037 | 0.865 |
| MRVI1 | Lateral E/eʹ | 0.393 | 0.039 | 0.417 | 0.020 | 7.97E-05 | 0.141 | 0.520 |
| OLAH | Lateral E/eʹ | 0.504 | 0.006 | 0.356 | 0.049 | 3.03E-05 | 0.145 | 0.510 |
| *OR52N4* | *Septal E/eʹ* | *-0.377* | *0.048* | *-0.433* | *0.015* | *7.56E-05* | *-0.434* | *0.038* |
| *PROSC* | *Lateral E/eʹ* | *0.454* | *0.015* | *0.457* | *0.010* | *1.41E-05* | -0.214 | 0.326 |
| RNF130 | Lateral E/eʹ | 0.446 | 0.017 | 0.385 | 0.033 | 5.82E-05 | -0.132 | 0.548 |
| RNF144B | Lateral E/eʹ | 0.414 | 0.028 | 0.485 | 0.006 | 1.53E-05 | -0.051 | 0.818 |
| SH3PXD2B | Lateral E/eʹ | 0.389 | 0.041 | 0.383 | 0.034 | 1.51E-04 | -0.153 | 0.487 |
| *SLC16A2* | *Septal E/eʹ* | *0.384* | *0.044* | *0.378* | *0.036* | *1.75E-04* | *0.396* | *0.052* |
| *SULT2B1* | *Septal E/eʹ* | *0.418* | *0.027* | *0.385* | *0.032* | *9.30E-05* | *0.403* | *0.051* |
| TBX2 | Septal E/eʹ | 0.399 | 0.036 | 0.457 | 0.010 | 3.45E-05 | 0.121 | 0.582 |
| UBTD2 | Lateral E/eʹ | 0.456 | 0.015 | 0.395 | 0.028 | 4.16E-05 | -0.114 | 0.604 |
| ZNF185 | Lateral E/eʹ | 0.379 | 0.046 | 0.367 | 0.042 | 2.23E-04 | -0.225 | 0.302 |

Transcripts in italics met the criteria for further validation in a murine SCD model. Error bars indicate standard error.

| **Supplementary Table 2: Normalized mRNA expression levels in murine myocardial tissue from mice with and without SCD.** | | | | | | | | | | | | | |
| --- | --- | --- | --- | --- | --- | --- | --- | --- | --- | --- | --- | --- | --- |
| **ADAM9** | | **FUCA2** | | **SULT2B1** | | **PROSC** | | **IL18** | | **SLC16A2** | | **OLFR658 (OR52N4)** | |
| HbAA | HbSS | HbAA | HbSS | HbAA | HbSS | HbAA | HbSS | HbAA | HbSS | HbAA | HbSS | HbAA | HbSS |
| 1.27 | 0.90 | 1.18 | 0.94 | 0.87 | 0.41 | 1.07 | 0.94 | 1.16 | 1.56 | 1.05 | 2.05 | 1.35 | 0.74 |
| 1.06 | 1.14 | 1.36 | 1.42 | 0.57 | 0.74 | 0.98 | 0.94 | 0.91 | 1.07 | 1.31 | 1.61 | 0.95 | 1.63 |
| 0.84 | 1.22 | 0.90 | 1.68 | 0.68 | 1.46 | 0.94 | 0.82 | 0.86 | 1.47 | 0.99 | 1.90 | 1.13 | 0.79 |
| 0.84 | 1.37 | 0.89 | 1.77 | 1.20 | 1.64 | 0.94 | 0.87 | 1.12 | 1.51 | 0.86 | 1.84 | 0.77 | 0.98 |
| 0.90 | 1.29 | 1.04 | 1.27 | 1.26 | 0.95 | 0.96 | 0.71 | 0.63 | 0.91 | 0.79 | 1.24 | 0.80 | 0.54 |
| 1.22 |  | 0.90 |  | 1.45 |  | 0.89 |  | 0.61 |  | 1.09 |  |  |  |
| 0.86 |  | 0.74 |  | 0.97 |  |  |  |  |  | 0.92 |  |  |  |

**Calculations for probability of a candidate gene meeting association thresholds by chance:**

Bonferroni adjustment for 12,549 individual tests:

0.05^*^/12,549 = 3.98 x 10^-6^

Probability of replication, validation, and eQTL association (all in the same direction):

0.05^*^ x 0.05^&^ x 0.5^#^ x 0.05^‡^ x 0.5^†^ x 1.4^ǂ^ x 0.5^¥^ = 1.75 x 10^-6^

^*^ = Threshold for Discovery Cohort

^&^ = Threshold for Replication Cohort

^#^ = Probability that replication association is in the same direction

^‡^ = Threshold for validation in murine myocardial tissue

^†^ = Probability that validation association is in the same direction

^ǂ^ = 0.05/28 = Probability of SNP being associated with E/eʹ for 28 SNPs – the highest number of SNPs tested in a gene)

^¥^ = Probability that a SNP association is in the same direction
